# An ecohydrological approach to the river contamination by PCDDs, PCDFs and dl-PCBs – concentrations, distribution and removal using phytoremediation techniques

**DOI:** 10.1038/s41598-019-55973-3

**Published:** 2019-12-17

**Authors:** M. Urbaniak, E. Kiedrzyńska, A. Wyrwicka, M. Zieliński, E. Mierzejewska, M. Kiedrzyński, K. Kannan, M. Zalewski

**Affiliations:** 1 European Regional Centre for Ecohydrology of the Polish Academy of Sciences, Tylna 3, 90-364 Lodz, Poland; 2 Department of Applied Ecology, Faculty of Biology and Environmental Protection, University of Lodz, Banacha 12/16, 90-237 Lodz, Poland; 3 Wadsworth Center, New York State Department of Health, Empire State Plaza, Albany, NY 12201-0509 USA; 4 Department of Plant Physiology and Biochemistry, Faculty of Biology and Environmental Protection, University of Lodz, Banacha 12/16, 90-237 Lodz, Poland; 5 Nofer Institute of Occupational Medicine, Teresy 8, 91-348 Lodz, Poland; 6 Department of Geobotany and Plant Ecology, Faculty of Biology and Environmental Protection, University of Lodz, Banacha 12/16, 90-237 Lodz, Poland

**Keywords:** Environmental biotechnology, Abiotic, Environmental impact

## Abstract

The levels of polychlorinated dibenzo-*p*-dioxins (PCDDs), polychlorinated dibenzofurans (PCDFs) and dioxin-like polychlorinated biphenyls (dl-PCBs) in the Pilica River and Sulejów Reservoir were found to be 46% higher during the flood season than during stable flow periods. In addition, PCDD/PCDF and dl-PCB mass loads increased by 5- to 12-fold and by 23- to 60-fold for toxic equivalency (TEQ) during flooding. The Sulejów Reservoir was found to play a positive role in reducing PCDD, PCDF and dl-PCB transport within the study period, with reductions ranging from 17 to 83% for total concentrations, and 33 to 79% for TEQ. Wastewater Treatment Plants (WTPs) were not efficient at mass concentration removal, with small displaying the least efficiency. WTPs discharge pollutants into the aquatic environment, they also produce sludge that requires disposal, similar to reservoir sediments. Sludge- or sediment-born PCDDs, PCDFs and dl-PCBs may be removed using phytoremediation. The cultivation of cucumber and zucchini, two efficient phytoremediators of organic pollutants, on polluted substrate resulted in a mean decrease in PCDD + PCDF + dl-PCB TEQ concentrations: 64% for cucumber and 69% for zucchini in sludge-amended soil, and by 52% for cucumber and 51% for zucchini in sediment-amended soil.

## Introduction

A river catchment is a highly-complex ecological system. Hydrological pulses facilitate the transport of nutrients, mineral and organic matter along the river continuum^1^. The levels of anthropogenic pollutants in the river basin, including toxic organic compounds and endocrine disruptors are increased by human activity. As they are positioned at the lowest points in the landscape, rivers often accumulate pollutants transported via atmospheric deposition, runoff from urban and agricultural areas or inflow through point sources^2–5^.

Being so susceptible to pollutants, riverine ecosystems are subject to strict monitoring, protection and rehabilitation. For example, Poland is obliged to implement the objectives of the EU Water Framework Directive (2000/60/EC) regarding community action for water policy, and the chemical and biological status of water resources. Good river water status can be achieved by eliminating or reducing emissions from the most serious pollutants. To this end, the Water Framework Directive (2000/60/EC) and the Directive of the European Parliament and Council (2013/39/EU of 12 August 2013 amending Directive 2000/60/EC and 2008/105/EC) regarding priority substances in the field of water policy (Directive EQS) list 45 substances representing a serious threat to aquatic environments and to humans: these need to be removed from aquatic environments. Three groups of pollutants on the list are PCDDs, PCDFs and dl-PCBs.

One of the greatest sources of PCDDs, PCDFs and dl-PCBs in the river is human activity in its catchment. Depending on the catchment land use type: agricultural vs urban/industrial, the main sources can include farming and associated to this impairment of water circulation as well as the intensification of erosion and accelerated transport of micropollutants to water bodies. Runoff of deposited pollutants from the city space or discharge of untreated or insufficiently treated wastewater of municipal and industrial origin, in turn, mainly contributes to pollution of rivers located in urban/industrial catchments^6,7^. Extremely low water solubility of PCDDs, PCDFs and dl-PCBs allows them to bind to the organic and mineral particles in the river; in this bound form, they are transported along the river continuum and deposited downstream. After deposition, they gradually accumulate in sediments, which serve as long-term sources of pollutants.

To ensure the good health of river users, to secure the quality of the environment, and to achieve the goals of the EU strategy, it is necessary to reduce the degree of pollution by these compounds. The principles of ecohydrology^2–5^, indicate that a thorough assessment of the condition of the environment is required before the degree of contamination can be reduced or reversed within the catchment scale, together with a quantification of the environmental problem. A broad spectrum of analyses is performed to determine the concentration of the given pollutant and its toxicity with regard to the individual components of the ecosystem (*identification of threats*); the results would clarify the interactions and processes that evaluate the levels of pollutants (*analysis of cause-effect relationships*). The findings are then used to improve the quality of the environment (*developing methods and tools for reduction of identified threats*).

Hence, this study not only assesses the concentration and toxicity of PCDDs, PCDFs and dl-PCBs in the riverine environment, but more importantly, also defines their roles in the ecosystem and identifies the processes determining their concentrations. Its findings indicate opportunities for their safe removal. The contaminated sediment can be remediated by the development of environmental biotechnologies, such as microbial remediation and phytoremediation: these being the removal or detoxification of pollutants with the use of microbes or plants.

**Figure 1:**
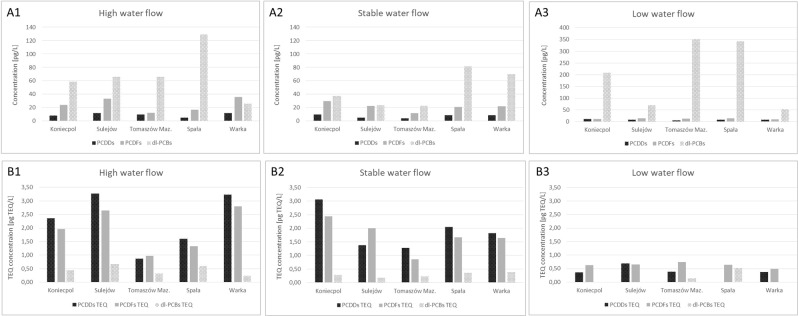
The role of hydrology (high, stable and low water flow) on the spatial dynamics of PCDDs, PCDFs and dl-PCBs in the Pilica River along the river continuum from upland to downstream: **A1–A3** – total concentrations; **B1–B3** – TEQ concentrations.

Hence, the main objectives of this paper were to identify the threats associated with the presence of PCDDs, PCDFs and dl-PCBs in the Pilica River ecosystem (Poland) and to determine the factors influencing their concentrations; this was achieved by assessing: 1) the influence of the dam reservoir on contaminant migration, 2) hydrological (flow) conditions and 3) the role of point sources of pollution in the river catchment on the concentrations, toxicity (measured as TEQ) and distribution of PCDDs, PCDFs and dl-PCBs along the river continuum. In addition, the study (4) evaluated the potential of selected *Cucurbitaceae* for the removal of studied pollutants from river/reservoir bottom sediments and wastewater treatment plant sludge (phytoremediation) (Fig. 1SI).

## Results and Discussion

### The impact of the dam reservoir on PCDD, PCDF and dl-PCB reduction in the Pilica River

The River Continuum Concept^1^ indicates that ecological processes occurring along the course of a river demonstrate continuity and geomorphological, biological and chemical gradients. The same is true for pollutants migrating along the river system: their levels should be interpreted across a broad space-time context which considers both the role of the catchment and the river itself, as well as the impact of present and past sources of pollution.

PCDD, PCDF and dl-PCB concentrations are known to increase along a polluted river, reaching a peak in the mouth section^6,8–13^.

The transport of pollutants along a river can be prevented by their sequestration in sediments from rivers and reservoirs. The presence of reservoirs along a river favours pollutant sedimentation and accumulation by slowing its flow, lengthening water retention time and introducing large loads of matter into the water. Reservoirs therefore improve water quality below dams by interrupting their transport and acting as traps for pollutants bound to inorganic and organic matter^13–20^.

Our findings confirm that Sulejów Reservoir plays a key role in lowering the concentrations of the target compounds along the river. The total and TEQ concentrations of dl-PCBs were much lower in the sediments noted below the dam (Tomaszów Maz. sampling station) than in the samples collected above the reservoir (Sulejów sampling station, Fig. 1): total concentration fell by 83%, from 13.7 to 2.90 ng/kg, and TEQ concentration fell by 79%, from 0.530 to 0.110 ng TEQ/kg (Table 1). The Sulejów Reservoir was also found to reduce the TEQ concentrations of PCDDs/PCDFs in the bottom sediments by up to 49%, while the total concentration increased by 27%, mostly due to a higher OCDD congener concentration (Table 1).

**Table 1:** The role of the Sulejów Reservoir in the reduction of PCDDs/PCDFs and dl-PCBs transported by the Pilica River continuum.

Total concentration				
	Compound	Inflow to reservoir	Outflow from reservoir	% reduction
Bottom sediments [ng/kg d.w]	PCDDs/PCDFs	176	224	*−27*
	dl-PCBs	13.7	2.90	**83**
River water [pg/L]	PCDDs/PCDFs	50.5 +/− 29.3	35.7 +/− 24.9	**30**
	dl-PCBs	53.3 +/− 18.2	44.5 +/− 17.4	**17**
Load [mg/day]	PCDDs/PCDFs	341 +/− 368	128 +/− 136	**62**
	dl-PCBs	401 +/− 387	321 +/− 342	**20**
**TEQ concentration**				
Bottom sediments [ng/kg d.w.]	PCDDs/PCDFs	1.38	0.710	**49**
	dl-PCBs	0.530	0.110	**79**
River water [pg/L]	PCDDs/PCDFs	4.00 +/− 1.60	1.80 +/− 0.300	**55**
	dl-PCBs	0.300 +/− 0.200	0.200 +/− 0.100	**33**
Load [mg/day]	PCDDs/PCDFs	42.3 +/− 45.0	10.1 +/− 10.9	**76**
	dl-PCBs	28.8 +/− 30.7	13.0 +/− 13.8	**55**

Negative value indicates increase in the concentration

An analysis of river water at the inflow and outflow from the Sulejów Reservoir also revealed 17 and 33% reductions in total and TEQ dl-PCB concentration, and 30 and 55% reductions in total and TEQ PCDD/PCDF content. In addition, considerable reductions in dl-PCB and PCDD/PCDF loads were observed: 20 (total concentration) and 55% (TEQ) decreases were found for dl-PCBs, while 62 (total) and 76% (TEQ) reductions were found for the PCDD/PCDF loads (Table 1).

This considerable reduction of the total and TEQ concentrations of the studied compounds is related to the limnological characteristics of the reservoir. Total water exchange occurs typically nine times per year. Although the mean water retention time in the Sulejów Reservoir is 42 days, measured over its lifetime, this value changes over the course of the year: May 2010–10.4 days, September 2010–14 days, June 2012–56.6 days. The increased retention time during the summer period affects the water flow and reduces its lifting force, thus enhancing the sedimentation of the coarse particulate matter and fine particles on which PCDDs/PCDFs and PCBs are adsorbed. The coarser sediments tend to be found in the upper, riverine, part of reservoirs, while the fine particles accumulate in the lower, limnological, part. This may lead to periodical retention of up to 97% of the studied compounds^21^.

Despite the above, it is important to underline that under certain conditions the reservoir may act also as source of pollutants releasing PCDD, PCDF and dl-PCB accumulated in its sediments back to the water column. Such situation can happen under reduced external pollutants discharge – in this case pollutants accumulated on the bottom of reservoir may be released back to the water column. Moreover, high, turbulent flow may lead to resuspension of the accumulated sediments and associated pollutants. Since the sedimentation and deposition processes are the main factors affecting the reductions of studied compounds in the reservoir water, turbulent flow reduces the sediment deposition and shapes the water body quality in the reservoir and below the dam^19^.

Although the results obtained within our study highlight the positive influence of the Sulejów Reservoir with regard to pollution, they also clearly show the problems faced by the Pilica River and it reservoir associated with PCDD, PCDF and dl-PCB pollution. As the quality of the sediment and water in the reservoir is determined by processes occurring across the whole catchment-river-reservoir system, the final dynamics and distribution of PCDDs/PCDFs and dl-PCBs are shaped by a complex interaction between the level of pollution and the existing biogeochemical (biological, chemical and physical) and hydrological processes related to the water cycle within a specific system. Therefore, further analyses were performed to determine the impact of hydrological conditions and point sources of pollution on the concentrations, toxicity and distribution of PCDDs/PCDFs and dl-PCBs along the Pilica River continuum.

### The impact of hydrological conditions on the concentration, toxicity and the distribution of PCDDs, PCDFs and dl-PCBs along the river continuum

Global climate change has a quantitative and qualitative effect on water resources, which is reflected in disturbances in the hydrological cycle and water flow in rivers. It is also likely that the frequency and intensity of the flood and drought periods will also be distorted, and that this will affect the economic and sociological development of regions. According to Milly *et al*.^22^, the increased global water circulation and level of rainfall associated with warming of the climate raises the risk of flooding by generating a greater amount of surface runoff^23–27^. In Poland, more specifically the South Baltic catchment area, where the annual rainfall is 600 mm and 70% of the surface is covered with agricultural land, the climate scenarios of Baltic Marine Environment Protection Commission - Helsinki Commission (HELCOM) predict as much as 70% increases in river flow during the winter season and around 50% reductions during the summer season^28^. Such drastic changes will dramatically increase the chance of extreme floods and droughts, and pose a serious threat to sustainable development. Since water is the primary medium responsible for the transport of matter, nutrients and pollutants from the catchment, their concentrations and loads will change dramatically in inland waters. It is worth to note that Polish emissions of PCDDs/PCDFs to air from residential combustion contributed to more than 70% of the total load from this source within the Baltic Sea catchment. Emissions to air are indicated to be an important source of the loads of these compounds to land and water ecosystems via dry and wet deposition and their further flushing to rivers during intensive rains.

Therefore, our study examined how the hydrological conditions prevailing in the Pilica River and its catchment influence the mass concentration, TEQ and distribution of PCDDs/PCDFs and dl-PCBs along its continuum. The study was conducted in three different sets of hydrological conditions occurring in the river: a period of extreme flow during spring flooding, one of stable flow and another of low flow during the dry season.

Matter, nutrients and pollutants are flushed out from the catchment area by flood waves, and transported to the river bed, particularly during the early flood stage^29,30^. This is confirmed by our current findings, indicating that hydrological conditions prevailing in the Pilica River (see Table 2SI) have a strong influence on the PCDD/PCDF and dl-PCB concentrations. The mean concentrations recorded for all sampling stations during the flood season were 46% higher than those observed during the period of stable flow (103 ng/L vs. 55.6 pg/L). However, the values noted at low water flow (224 pg/L) were twice as high as those measured for high water flow and four-times higher than during stable water flow. These findings were strongly influenced by the high dl-PCB concentrations observed at low water flow (Fig. 1). The total concentrations of PCDDs/PCDFs at high water flow (33.6 pg/L) were 16% higher than during stable conditions (28.3 pg/L) and 44% higher than during low water flow (18.7 pg/L). In the case of total TEQ values, the mean concentrations noted at high water flow (4.60 pg TEQ/L) were 15% higher than those observed during stable flow (3.90 ng TEQ/L) and 76% higher than values at low water flow (1.10 pg TEQ/L) (Fig. 1). Despite the strong influence of hydrological conditions on the noted values, PCDDs/PCDFs and dl-PCBs concentrations have not been related to the content of mineral, organic and total matter in the water samples nor the biogenic compounds such as phosphorus and nitrogen (Table 2SI).

Our findings also showed a 46% increase in total PCDD level (from 8.29 to 12.1 pg/L) and a 52% increase in total PCDF concentration (from 23.5 to 35.7 pg/L) along the river course at high water level. While, at stable and low water flow the levels of these compounds decreased respectively of 9.5% (from 9.72 to 8.80 pg/L) and 26% (from 10.5 to 7.68 pg/L) for PCDDs, and 26% (from 29.2 to 21.5 pg/L) and 18% (from 10.8 to 8.8 pg/L) for PCDFs. The opposite pattern was observed for dl-PCBs, i.e. concentration decreased by 55% (from 58.8 to 26.4 pg/L) at high flow and increased by 85% (from 37.60 to 69.80 pg/L) at stable water flow. Low water level was again associated with a 74% reduction of dl-PCB values (from 208 to 52.8 pg/L) (Fig. 1). The total TEQ values also decreased along the river course during stable (a decrease of 33%) and low water flow (a decrease of 13%); however, the level increased by 32% during high water level (Fig. 1).

**Figure 2:**
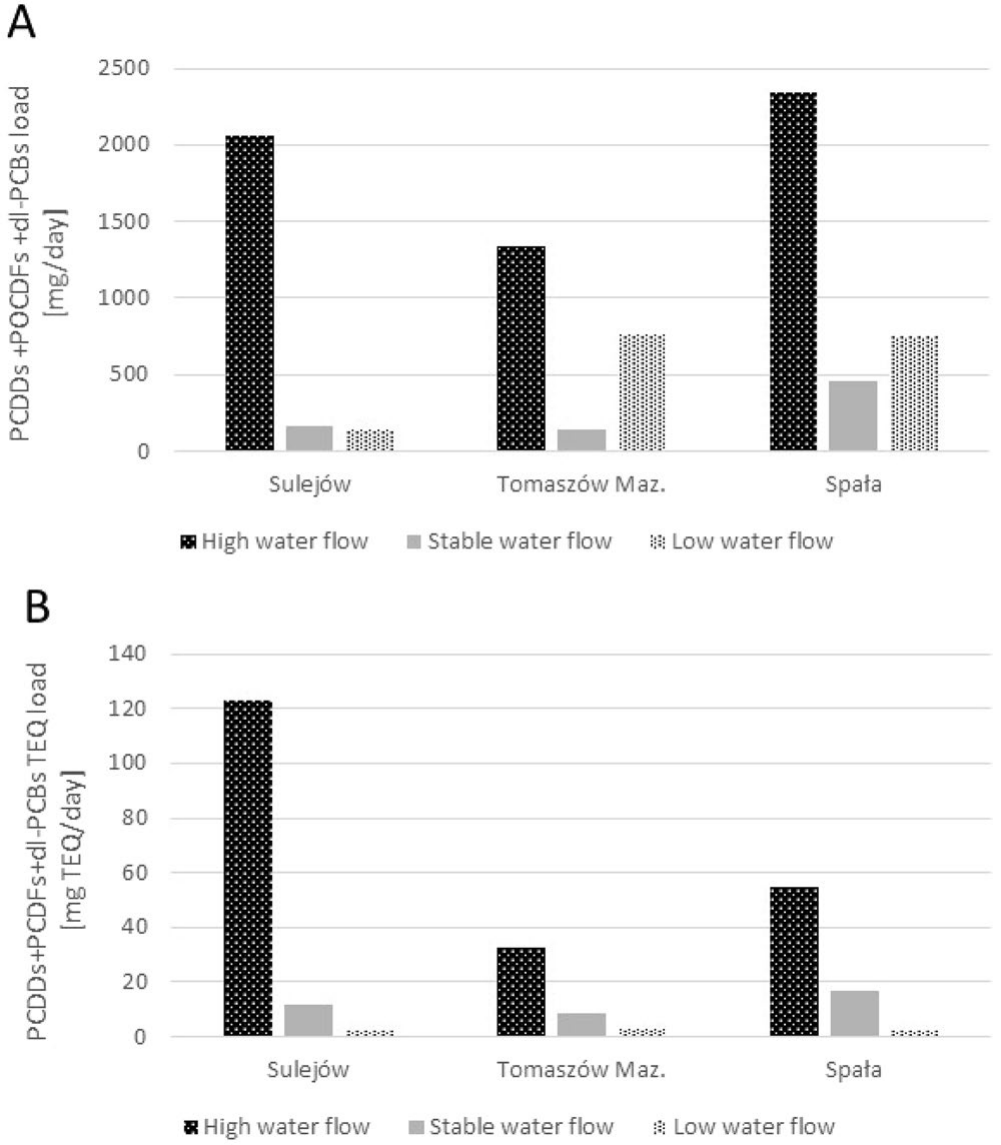
The role of hydrological factors (high, stable and low water flow) on total PCDD + PCDF + dl-PCB and total TEQ loads transported along the continuum of the Pilica River at Sulejów, Tomaszów Maz. and Spała river gauge stations.

In addition to changes in the total concentration and TEQ values, the PCDD, PCDF and dl-PCB profiles also changed periodically in response to hydrological fluctuations. The contribution of PCDDs in the total PCDD + PCDF + dl-PCB content was the most consistent at all five sampling stations during stable flow conditions, ranging from 8 to 13%. Greater variation was observed during high and low water flow, with a higher PCDD share observed at high flow (3–16%) and a lower share at low water flow (1–11%) (see Fig. 2SI). The proportion of PCDFs ranged from 11 to 48% at high flow, from 19 to 43% at stable conditions, and from 3 to 15% at low flow conditions. Such small percentage of both PCDDs and PCDFs in the total values noted at low flow was determined the high contribution of dl-PCBs being from 76 to 96%, while at high and stable flow ranged from 36 to 86% and from 46 to 74%, respectively (see Fig. 2SI). PCDDs constituted the greatest proportion of TEQ concentration at high flow, ranging from 40 to 52% of total TEQ; this value ranged from 39 to 54% at stable flow and from 0 to 51% at low flow. In contrast, PCDFs constituted between 38 and 45% at high flow, between 36 and 56% at stable flow, and 48 to 63% at low flow (Fig. 2SI). The smallest contribution to total TEQ was noted for dl-PCBs, ranging from 4 to 16% at high flow, from 5 to 10% at stable flow, and from 1 to 44% at low water flow (see Fig. 2SI).

The altered hydrological conditions also have an influence on pollutant loads. Our findings demonstrate that total transported PCDD + PCDF + dl-PCB load was five- to 12-times greater during flood conditions than during stable water flow, and two- to 15-fold greater than during low water flow (Fig. 2). Much greater differences were observed for the TEQ loads: the TEQ load transported during high water flow was as much as 23-fold (at the Spała water gauge) and 60-fold higher (at Sulejów) than observed during low flow. The TEQ loads calculated for stable water flow were three-fold to 10-fold lower that those noted for high water flow, and three-fold to seven-fold higher than for low flow (Fig. 2).

Such significant differences in the concentrations of studied compounds, and their loads, are predominantly influenced by the hydrological conditions prevailing in the river itself. One important hydrological influence is turbulent flow, which causes the resuspension of bottom sediment and its associated pollutants. It should be also mentioned that the studied compounds are deposited in the catchment area by various sources, including wet and dry atmospheric deposition, and by agricultural activities such as the use of plant protection products known to contain studied pollutants or being their precursors in the environment^31,32^. During rain events, the pollutants deposited in the catchment can be flushed to the rivers. As the Pilica River catchment is not a homogenous in term of its land use type (Table 1SI), different subcatchments of varied land cover may have different impact on the quality of river water. As depicted in Table 1SI the agriculture is the dominant form of land use within all subcatchemnts and thus may have an intrinsic impact of the noted values. Nevertheless, two subcatchments (3 and 4) are characterized with elevated percentage of urban and industrial areas that may additionally influence on the quoted concentrations. The level of pollution is further exacerbated by the absence of ecotones that could otherwise diminish the load of pollutants entering from diffuse sources; hence, high concentrations of the studied compounds are observed during extreme flow conditions preceded by intense rains^7^.

In contrast to flood, stable flow conditions promote sedimentation and deposition of pollutants in the river and reservoir sediments, thus reducing the concentrations of studied compounds transported via water. Moreover, they also reduce catchment runoff, thus preventing pollutants from being scoured from the river catchment and transported to the river ecosystem, as was demonstrated during flooding.

On the other hand, however, due to the low volume of river water, low flow conditions are characterised by higher concentrations of pollutants associated with human activity which are continually introduced into the river, for example, wastewater discharges^7^. Hence high concentrations of dl-PCBs were observed in the samples collected at the Spała and Tomaszów Maz. sampling stations (Fig. 1): the catchment area with highest share of urban areas (Table 1SI), where the largest WTPs are located.

To conclude, an analysis of hydrological conditions found water flow to have a decisive role on the loads of the target compounds, resulting in the observed values being 23–60 times greater during high flow than during low flow. Future studies should more closely examine the flood phases that transport the highest concentrations of PCDDs, PCDFs and dl-PCBs with the aim of reducing their levels; this could be achieved through the retention of the flood wave on floodplain terraces, or in reservoirs based on specific biological structures designed to enhance the biodegradation potential of the studied compounds using biological and phytoremediation processes^33–40^. Skłodowski *et al*.^41^ examine the role played by naturally-occurring biological and phytoremediation processes taking place in the river valley in the reduction of these compounds, and report a 49% reduction of TEQ concentrations of dl-PCBs in sediments within a 40-km natural meandering section of the Pilica River covered with a riparian vegetation community.

We observed strong reductions of total PCDD + PCDF + dl-PCB concentration (33%) and TEQ concentration (38%) during stable flow in the river section between the Koniecpol and Sulejów sampling stations (100.4 km length of river section): a sector covered by natural vegetation (forests cover 45.43% of the total subcatchment area, Table 1SI), dominated by riparian willow communities. These results indicate that the presence of natural floodplain terraces in river valleys significantly increases the rate of self-purification of the river^42,43^, and can reduce the transport of PCDDs/PCDFs and dl-PCBs along the river continuum. Similar findings have been noted by Bayley^44^, Burt *et al*.^45^, Barendregt^46^, Loeb and Lamers^47^. A greater knowledge of such relationships will allow a better understanding of the functioning of complex river ecosystems and enable their efficient management, thus minimizing the transport and accumulation of micropollutants in the sediments of rivers, reservoirs and estuaries.

### WTPs as source of PCDD, PCDF and dl-PCB pollution in the Pilica River

Point sources of pollution which continuously discharge contaminants to the aquatic ecosystem represent a serious threat to aquatic environments^20,48^. One example of these sources is that of outflows from WTPs.

With the increase of water consumption and its consequent increase of treated wastewater production/discharge, the question arises about the quality of the treated wastewater discharged to receivers, typically rivers. Routine monitoring of wastewater quality typically includes analyses of Biological Oxygen Demand (BOD_5_), Chemical Oxygen Demand (COD), nitrate, and phosphate^49^, however, this analysis alone does not fully reveal the risks posed by the introduction of treated sewage into rivers, as wastewater may also contain several unmonitored pollutants, including PCDDs, PCDFs and dl-PCBs.

It should be emphasized that no current national legislation defines the permitted concentrations of PCDDs, PCDFs and dl-PCBs in wastewater discharged into rivers. Only Article 41 of the Polish Water Law Act requires that the sewage introduced into the water, as part of regular or special use, cannot contain PCBs, but it does not regulate the limits for PCDDs + PCDFs. However, at EU level, Regulation (EC) No 166/2006 of the European Parliament and the Council (18 January 2006) concerning the establishment of a Register of European Pollutant Release and Transfer, and amending Council Directive 91/689/EEC and 96/61/EC (amended) does set such limits (Regulation EC No. 596/2009). It specifies that the maximum PCDD + PCDF TEQ dose introduced through WTPs into the water column cannot be greater than 0.0001 kg/year. However, smaller WTPs are not monitored for concentrations of PCDDs + PCDFs, as this provision applies only to the largest WTPs: those with a population equivalent (p.e.) of more than 100,000. It is therefore important to determine both the range and variability of the PCDD, PCDF and dl-PCB concentrations within treated wastewater outflows from WTPs, as well as their impact on river water quality.

**Figure 3:**
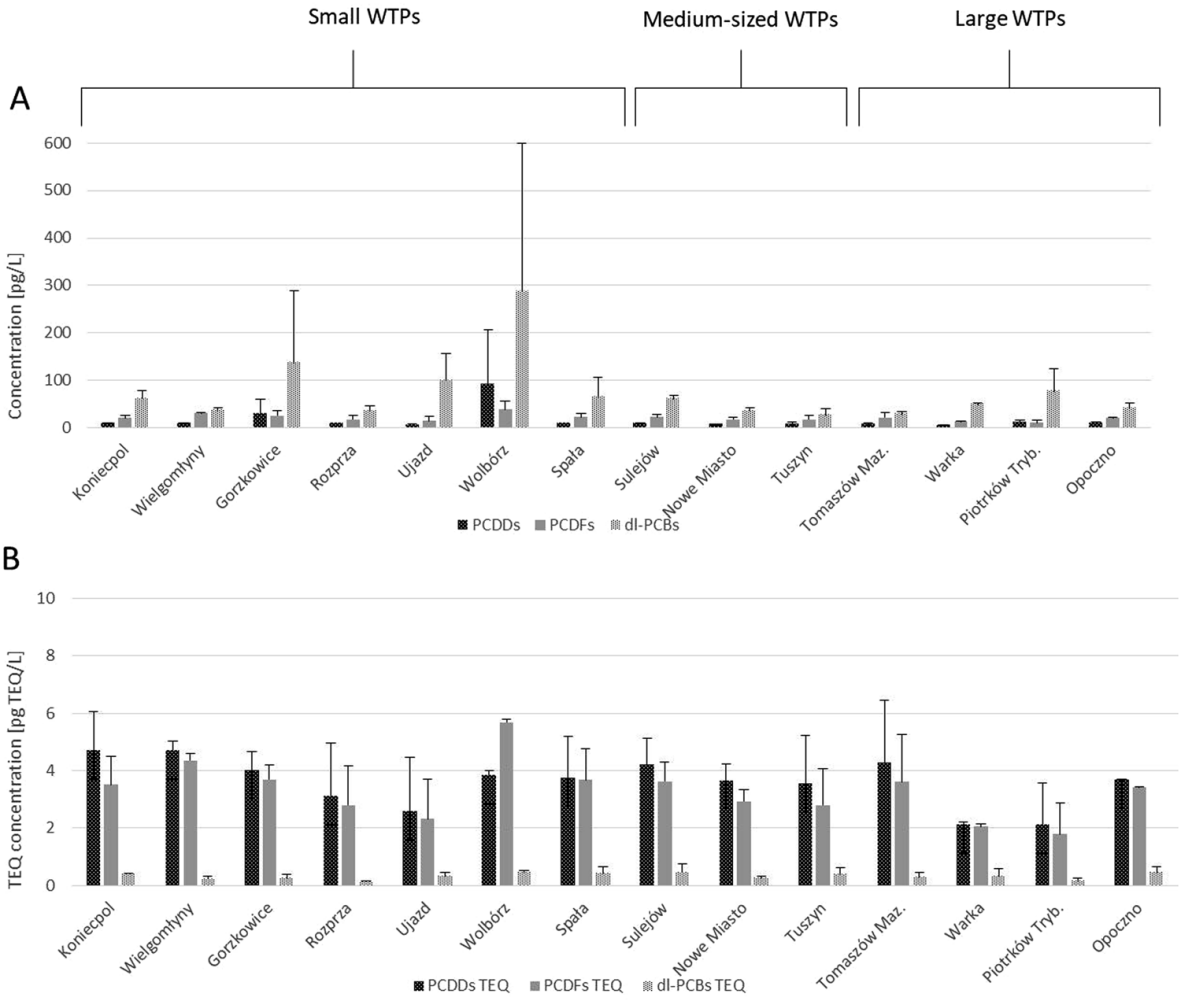
Concentrations of PCDDs, PCDFs and dl-PCBs in treated wastewater coming from particular WTPs in the Pilica River catchment (Poland) classified into three categories of size: small, medium-sized and large WTPs: (**A**) concentrations of PCDDs, PCDFs and dl-PCBs (n = 2) (**B**) TEQ concentrations of PCDDs, PCDFs and dl-PCBs (n = 2).

The effluents collected at the WTP outlets were found to contain 4.30 to 90.7 pg/L PCDDs, 10.6 to 38.9 pg/L PCDFs, and 51.4 to 287 pg/L dl-PCBs. The highest total concentration was noted in treated wastewater obtained from the small WTP in Wolbórz (417 pg/L), while the lowest was found in a medium-sized WTP in Tuszyn (51.4 pg/L) (Fig. 3). TEQ values ranged from 4.07 pg TEQ/L in the case of large WTP in Piotrków Tryb., to 9.98 pg TEQ/L from a small WTP in Wolbórz (Fig. 3).

**Figure 4:**
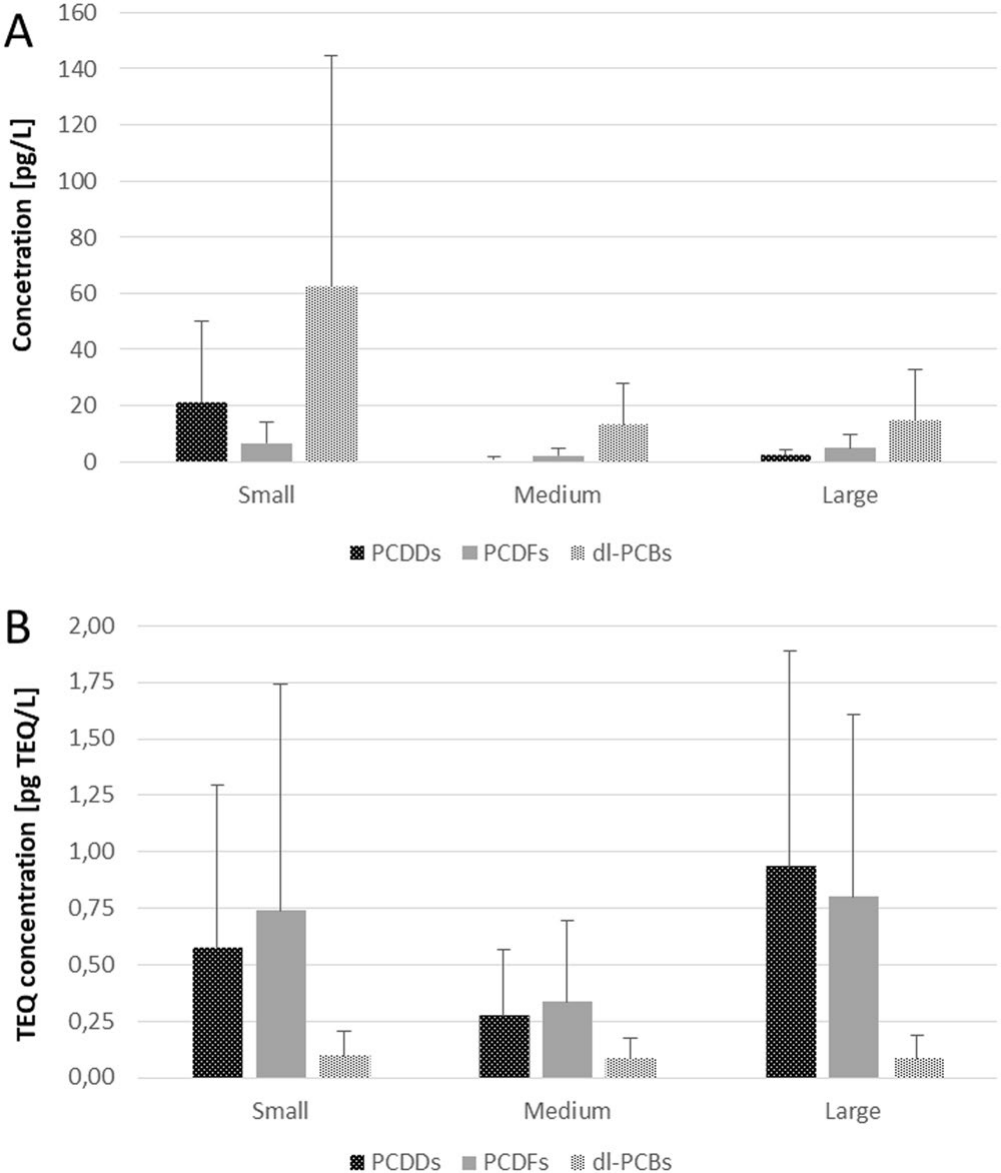
Mean concentrations of PCDDs, PCDFs and dl-PCBs in treated wastewater coming from the three groups of WTPs size: small (n = 14), medium-sized (n = 6) and large (n = 8) located in the Pilica River catchment: (**A**) mean (+/−SD) concentrations; (**B**) mean (+/−SD) TEQ concentrations.

Small WTP effluents were found to have higher concentrations of the studied compounds than those of the medium-sized and larger WTPs. The mean PCDD + PCDF + dl-PCB concentrations were found to be 88.0 +/− 125 pg/L for small WTPs, 16.5 +/− 21.8 pg/L for medium WTPs and 12.8 +/− 18.0 pg/L for large WTPs. Total PCDD concentration ranged from 0.910 +/− 2.36 pg/L, in the case of the medium-sized WTPs, to 21.2 +/− 28.8 pg/L for the small ones, with that of the large WTPs being 2.05 +/− 2.31 pg/L. A similar pattern was also observed for PCDFs and dl-PCBs (Fig. 4).

However, a different situation was observed in the case of mean PCDD + PCDF + dl-PCB TEQ. In this case, the highest values were observed in the large WTPs (1.81 +/− 1.87 pg TEQ/L), followed by the small WTPs (1.41 +/− 1.84 pg TEQ/L) and the least in the medium-sized WTPs (0.69 +/− 0.75 pg TEQ/L) (Fig. 4). Similar patterns were observed for PCDD TEQ, PCDF TEQ and dl-PCB TEQ, with the highest TEQ concentrations noted for the large WTPs, and the smallest for the medium-sized WTPs (Fig. 4). However, the TEQ values fell within a narrower range than the total concentrations.

**Figure 5:**
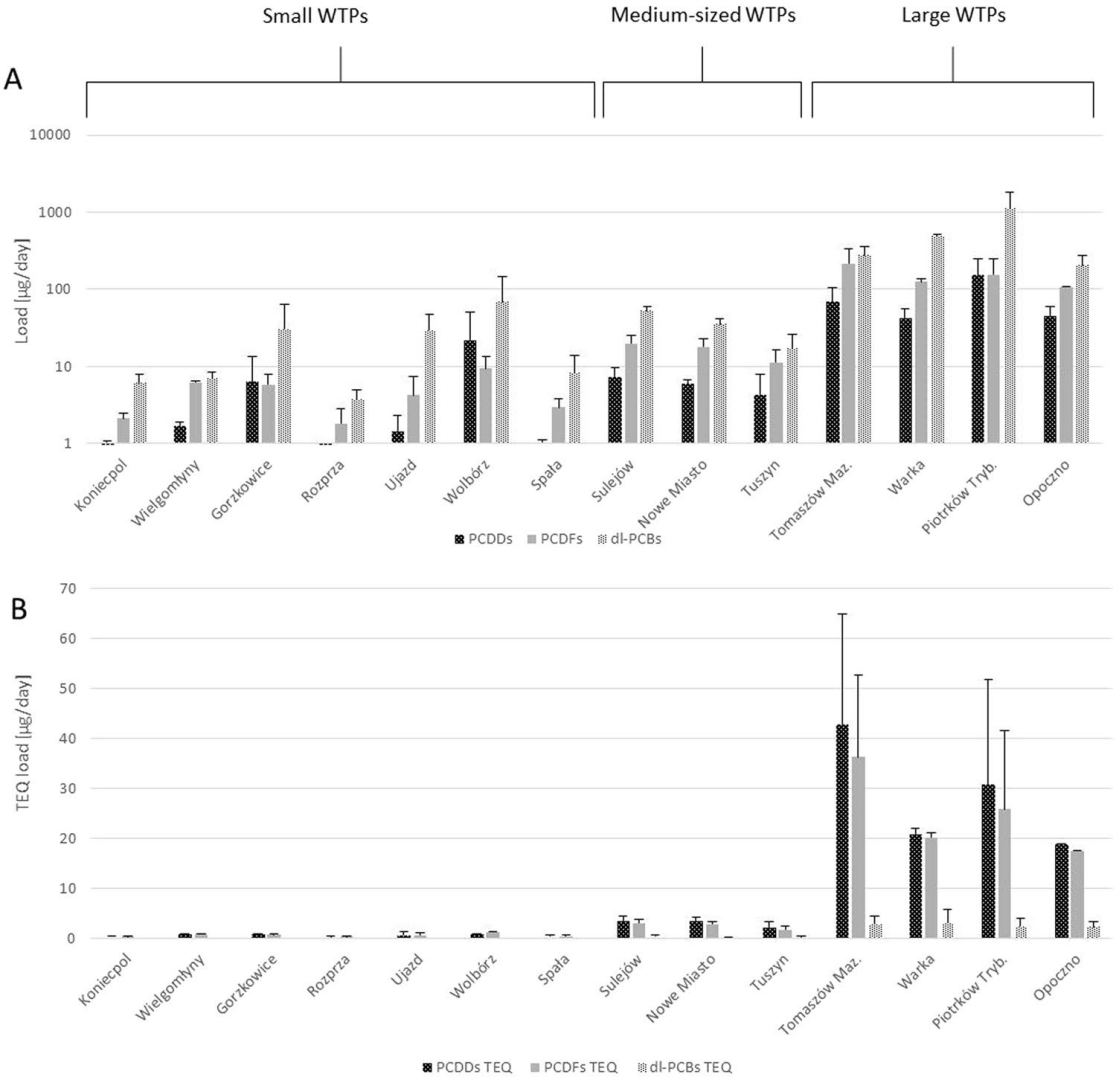
Mean daily loads (+/−SD) of PCDDs, PCDFs and dl-PCBs (n = 2) (**A**) and PCDDs TEQ, PCDFs TEQ and dl-PCBs TEQ (n = 2) (**B**) in outflowing wastewater from small, medium-sized and large WTPs located in the Pilica River catchment (Poland).

Larger WTPs and greater wastewater flow were also found to be associated with greater PCDD + PCDF + dl-PCB loads entering the rivers through their outflows. Among the small WTPs, the obtained PCDD loads ranged between 0.820 µg/day in Koniecpol to 21.9 µg/day in Wolbórz. PCDF loads were more uniform, ranging from 1.84 µg/day in Rozprza to 9.37 µg/day in Wolbórz, while Dl-PCB loads ranged from 3.75 µg/day in Rozprza to 69.2 µg/day in Wolbórz. Our findings indicate that Wolbórz WTP introduced the highest PCDD + PCDF + dl-PCB load into the river recipient (Fig. 5).

**Figure 6:**
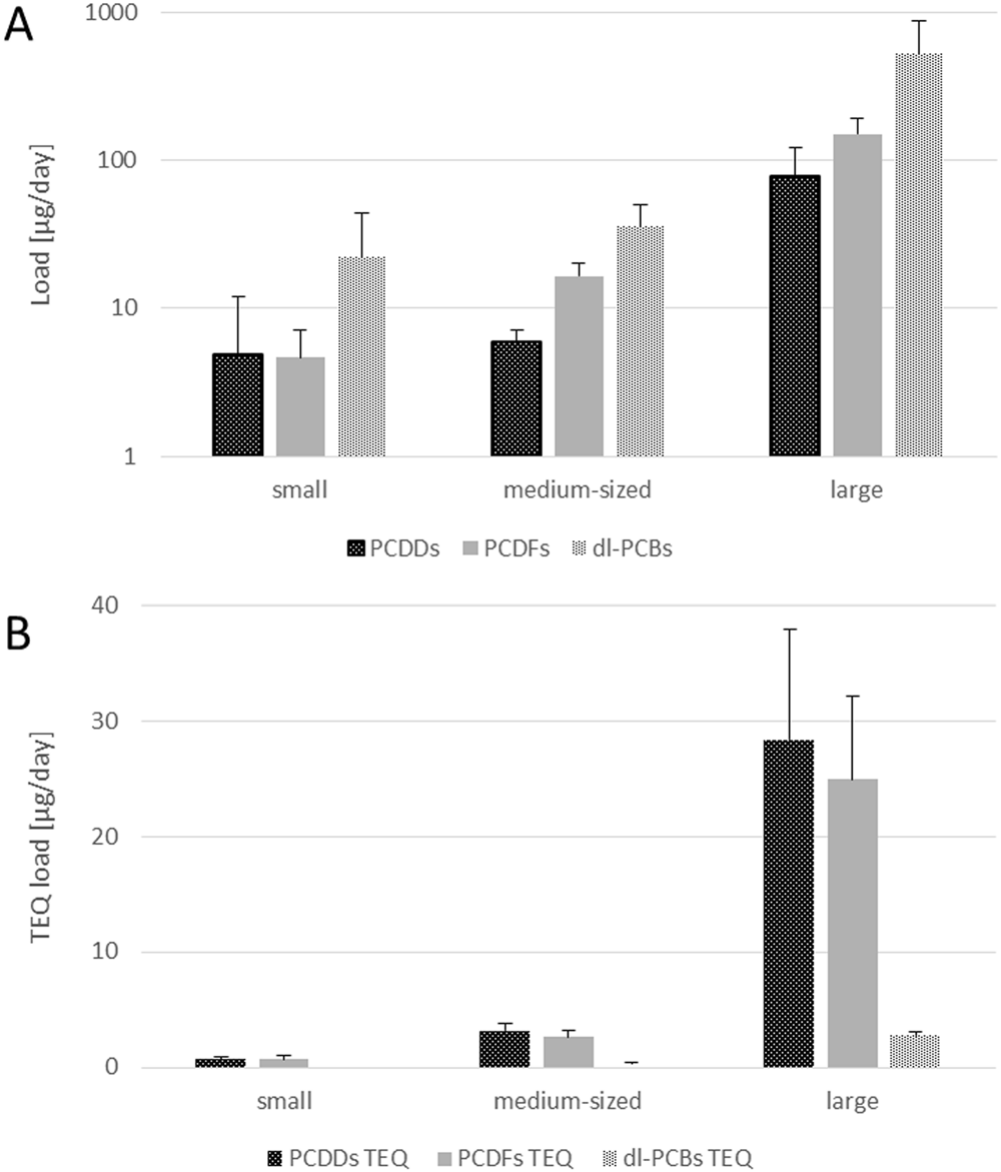
Mean (+/−SD) loads of PCDDs, PCDFs and dl-PCBs (**A**) and PCDDs TEQ, PCDFs TEQ and dl-PCBs TEQ (**B**) in outflowing wastewater from small (n = 14), medium-sized (=6) and large (n = 8) WTPs located in the Pilica River catchment.

In the case of medium-sized WTPs, the obtained loads were more unified; however, Tuszyn discharged the lowest amounts of the studied compounds, and Sulejów the highest (Fig. 6). PCDD levels ranged from 4.35 µg/day in Tuszyn to 7.33 µg/day in Sulejów WTP, PCDFs from 11.4 (Tuszyn) to 19.9 µg/day (Sulejów), and dl-PCBs from 17.2 (Tuszyn) to 53.2 µg/day (Sulejów) (Fig. 5).

Large WTPs discharged between 42.5 (Warka WTP) to 152 (Piotrków Tryb.) µg PCDDs per day, with PCDF loads ranging from 108 to 215 µg/day (Opoczno and Tomaszów Maz. WTP, respectively). The widest range of loads was observed for dl-PCBs, ranging from 204 µg/day (Opoczno WTP) to 1117 µg/day (Piotrków Tryb. WTP), and these constituted the greatest proportion of the load discharged from large WTPs (Fig. 5).

The highest load was generated by large WTPs. These discharge mean values of 748 +/− 404 µg of PCDD + PCDF + dl-PCBs to the river per day; while the small and medium WTPs discharged 31.6 +/− 30.1 and 57.6 +/− 19.4 µg per day, respectively. The same trends were seen for the sum totals of PCDDs, dl-PCBs and PCDFs (Fig. 6).

In the case of TEQ loads, the small WTPs introduced between 0.330 and 94.0 µg PCDD TEQ per day; 0.300 to 1.37 µg/day for PCDF TEQ, but only 0.0100 to 0.110 µg/day for dl-PCB TEQ. Rozprza WTP had the lowest total TEQ load (0.650 µg/day), and Wolbórz WTP the highest (2.40 µg/day). In contrast, the medium-sized WTPs demonstrated 2- to 3-fold higher TEQ loads than the small ones, ranging from 2.26 to 3.67 µg/day for PCDD TEQ, from 1.79 to 3.15 for PCDF TEQ and from 0.250 to 0.410 µg/day for dl-PCB TEQ. The smallest loads were noted for Tuszyn WTP and the highest for Sulejów WTP. The TEQ loads from the large WTPs were an order of magnitude higher, ranging from 18.9 to 43.0 µg/day for PCDDs, 17.5 to 36.3 µg/day for PCDFs, and 2.37 to 3.22 µg/day for dl-PCBs. The smallest loads were noted in Opoczno, and the highest in Tomaszów Maz. WTP (Fig. 5).

The mean TEQ loads increased together with WTP size, ranging from 1.45 +/− 0.58 µg/day for small WTPs, through 6.12 +/− 1.29 µg/day for the medium-sized WTPs, up to 56.1 +/− 16.8 µg/day for large WTPs (Fig. 6). The loads calculated for total PCDDs, total PCDFs and total dl-PCBs were found to have a similar pattern, with the lowest loads observed for small WTPs and the highest for large WTPs.

The present study is the first to examine the loads of PCDDs/PCDFs and dl-PCBs present in WTP effluent discharged to river recipients: most existing studies focus on such concentrations at different stages of the purification process, including the outflowing treated wastewater. PCDD/PCDF concentrations of 0.688 pg/L, and total PCB concentrations of 3.20 pg/L have been found in effluent from WTPs in Ostrava (Czech Republic)^50^, and PCDD/PCDF concentrations of 58.4 pg/m^3^ (0.0584 pg/L) in the outflow from Zabrze WTP^51^. Much higher concentrations were noted in the case of PCBs: Σ7 PCB level was found to be 250,000 pg/L in one WTP^52,53^ and PCB concentrations as high as 280,000 pg/L were found in the effluent of another^54^. Blanchard *et al*.^55^ report lower Σ13 PCB congener values ranging from 20 to 860 pg/L, while values of 1,400 pg/L^56^, and values between 1,000 and 6,000 pg/L^57^ have been reported elsewhere. Durell and Lizotte^58^ report PCB concentrations between 1,000 to 10,000 pg/L in effluent; however, these values have been found to be significantly lower elsewhere^59^ (500 and 4,100 pg/L).

Hence, the concentrations obtained in our present study are relatively low to moderate. However, such comparison is complicated by the lack of detailed results, especially those regarding concentrations and loads of PCDDs/PCDFs at WTP outlets.

Although our present results seem lower than those found in other studies, particularly regarding PCB content, they nevertheless indicate that the studied WTPs, especially the small ones, are still inadequate for the removal of these pollutants. This can be attributed to the outdated technology frequently used by the smallest WTPs, as well as the insufficient maintenance of the WTP and the purification process, and poor monitoring of effluent quality^20^. In turn, despite the relatively low concentrations, large plants typically introduce pollutant loads that are 24 and 13-fold greater than those of the small and medium-sized WTPs. The TEQ loads were found to be even greater for large WTPs, being 39-fold higher than those of the small WTPs, and nine-fold higher than the medium-sized WTPs. Such uncontrolled release in the WTP effluents may result in significant deterioration in the quality of river recipients^56,60,61^, in this case, the Pilica River and its tributaries. There is a clear need for more research into the fate of PCDDs, PCDFs and dl-PCBs during wastewater treatment; such knowledge will improve the quality of water ecosystems though the design of more effective methods for their removal (e.g. biofiltration systems, wetlands).

### Phytoremediation as an environmentally-friendly solution to remove PCDDs, PCDFs and dl-PCBs from contaminated sludge and bottom sediments

Our findings underline the need to mitigate the risks associated with inadequate wastewater treatment, and to ensure compliance with EU directives intended to ensure good ecological status for waters in Poland. According to the Directive of the European Parliament and Council (2013/39/EU), PCDDs, PCDFs and dl-PCBs are priority hazardous substances that should be eliminated at source, i.e the wastewater outlets of WTPs. One approach to remediation is based on the use of biofiltration systems: a hybrid Sequential Biofiltration System constructed at the treated wastewater outlet at Rozprza WTP removes 21% to 43% of PCB contaminants, with most elimination occurring in the vegetation area^62^.

Sequential sedimentation-biofiltration systems can also reduce the organic compound content in the outgoing wastewater. One example implemented in Asella (Central Ethiopia), enabled 70% removal of PCDDs, PCDFs and dl-PCBs: the pollutants were bound to sediments and deposited in the sedimentation zone, where they underwent photodegradation by direct sunlight; they were also degraded by microorganisms living in both the sedimentation and vegetation zones^63,64^. The system in Asella and a similar one on the Sokołówka River (Central Poland) have also demonstrated significant performance in the removal of nutrients and suspensions^5,63^.

In addition to discharging PCDDs, PCDFs and dl-PCBs into the aquatic environment, WTPs also produce sewage sludge as the end product. This sludge is typically stored within the area of the WTP or disposed of in landfills or incinerators. In Poland, over 31% of generated sludge is subject to storage and 14.5% to temporary storage (Statistical Yearbook of Environmental Protection, 2004–2009).

However, since 1 January 2015, the Polish Minister of the Environment (Journal of Laws of 2015, item 257) has prohibited the storage of sewage sludge characterized by the following parameters: total organic carbon content above 5% dry weight (d.w.), a loss on ignition of more than 8% d.w. and heat of combustion more than 6 MJ/kg d.w. There is, therefore, a need to develop cost-effective and environmentally-friendly methods of disposal. Fortunately, sludge is rich in organic matter and nitrogen, phosphorus, magnesium, calcium and sulphur, making it a valuable fertiliser for use in agriculture or in soilless land reclamation; however, despite its value in this regard this sludge contains a number of pollutants, not only PCDDs, PCDFs and dl-PCBs^65^.

There is also a need to utilize contaminated bottom sediments. As discussed above, the river ecosystems of Central Poland transport PCDDs, PCDFs and dl-PCBs, which are then partially deposited in the bottom sediments of reservoirs^13,19,66–68^ or possibly transported further along the river continuum to the Baltic Sea^11,12,69,70^.

Our findings suggest that it may be possible to use the contaminated reservoir sediments and sewage sludge as fertilizer. This is supported by the fact that they are generally rich in organic carbon and macronutrients

The research within this area is based on the ecohydrology concept, which proposes that ecosystem properties should be used to give access to “low-cost high technologies” by complementing engineering and technical solutions^41,71^. One of the key elements of ecohydrology is the improvement of the environment through the use of ecosystem biotechnologies derived using specialized knowledge about naturally-occurring processes such as phytoremediation.

The gourd family (*Cucurbitaceae*) have great potential to take up highly hydrophobic compounds such as PCDDs, PCDFs and dl-PCBs from soil and translocate them to the stems and leaves^72–77^. Recent studies indicate that this ability is related to the expression of the *MLP-GR3* gene, encoding a xylem protein of mass 17 kilodaltons^78^. Thanks to this considerable potential to sequester pollutants, the *Cucurbitaceae* represent an effective tool in the phytoremediation of PCDDs, PCDFs and dl-PCBs.

**Figure 7:**
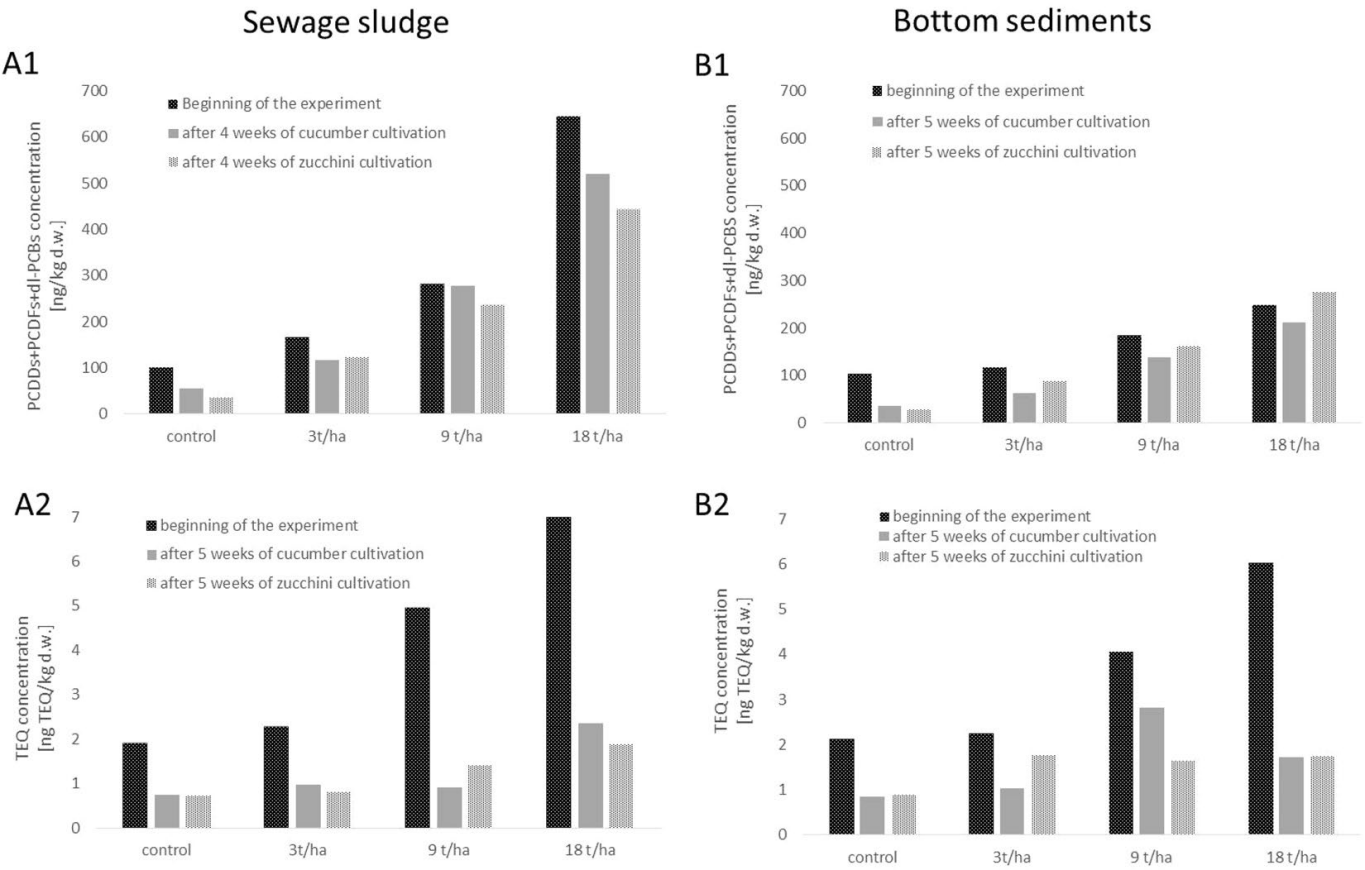
Reduction of total PCDDs + PCDFs + dl-PCBs and TEQ concentrations in soil amended with sewage sludge (**A1**; **A2**) or bottom sediments (**B1**; **B2**) using cucumber (*Cucumis sativus* L.) and zucchini (*Cucurbita pepo* L.).

Our results found sewage sludge to have a greater impact on PCDD + PCDF + dl-PCB contamination in the soil than bottom sediment. However, in both cases, the total and TEQ values of the treated soil increased with the applied dose of sludge or sediments. The application of 3, 9 and 18 t/ha sewage sludge resulted in total concentration increasing to 166, 279 and 630% and TEQ concentration increasing to 119, 260 and 367% of control values, respectively (Fig. 7). In the case of sediments, the used doses contributed to a smaller extent, with total values being 113, 178 and 239% of control values for the 3, 9 and 18 t/ha doses, respectively.

However, this effect was ameliorated by the use of cucurbits, i.e. cucumber and zucchini. The application of cucumber to sewage sludge-amended soil resulted in a 30, 1.3 and 19% (mean 17%) reduction of total values, and 43, 82 and 66% (mean 64%) reduction of TEQ values for the 3, 9 and 18 t/ha doses of sludge, respectively. The zucchini showed slightly greater reduction: 26, 16 and 31% (mean 24%) for total concentrations, and 64, 72 and 73% (mean 69%) for TEQ concentrations. In both cases, the smallest reductions were observed when the sludge dose of 9 t/ha was applied (Fig. 7).

In the case of bottom sediment-amended soil, cucumber cultivation reduced the total PCDD + PCDF + dl-PCB content by 46, 25 and 15% (mean 29%) and the TEQ concentrations by 54%, for the 3 t/ha, 31% for the 9 t/ha and 72% for the 18 t/ha doses (mean 52%). In contrast, zucchini cultivation resulted in reductions of 25, 13 and 11% (mean 16%) for total values, and 22, 59, and 71% (mean 51%) for TEQ values for the respective doses; these values were almost the same as observed for the cucumbers (Fig. 7).

Although the cucumber demonstrated greater reduction than zucchini in the samples amended with bottom sediments, the opposite was the case for sewage sludge-amended soil; similar results were obtained by Urbaniak *et al*.^75^. These differences may be attributed to differences in the pollutant composition of the two substrates: sewage sludge contains a number of other pollutants, such as heavy metals, which can adversely affect the health of the plants, and influence their remediation efficiency. However, sediments rich in iron and calcium have been found to be less toxic to the plant, possibly due to their ability to immobilize the metals^75^. In addition, cucumbers and zucchini are characterized by different properties and removal efficiencies against toxic organic pollutants^74^. Of the cucurbits, zucchini (*Cucurbita pepo* L.) has been found to be most efficient at accumulating organic compounds^77^; however, it has demonstrated more pronounced morphological (e.g. leaf blade damage) signs of toxicity than cucumber^79,80^. In our case, zucchini showed small protrusions of leaf surface immediately after application of sewage sludge. Also, chlorotic spots and bleaching appeared only on zucchini leaves, no such symptoms were observed for cucumber. The used amendments led also to different response of both plant species in term of their metabolic activity (e.g. activity of detoxifying enzymes). In the case of cucumber, our earlier study by Wyrwicka *et al*.^81^ demonstrated significant decrease in guaiacol peroxidase (POx) activity, being 70, 65 and 49% of the control value for 3, 9 and 18 t/ha of sewage sludge, respectively. Sediment application, in turn, had no significant influence on the enzyme activity. Similarly, glutathione S-transferase (GST), a multifunctional enzyme involved in xenobiotics metabolism in plants, demonstrated greater dependence on the sewage sludge - the enzyme increased significantly with the growing amount of sludge applied ranging from 148 to 172% of the control value, while sediments admixture had no statistically significant influence. Also, α-tocopherol, a lipophilic antioxidant, demonstrated different pattern among the plants cultivated in sewage sludge and sediment-amended variants; its concentration in the cucumber leaves increased with sewage sludge addition being statistically significant for the highest applied dose, while sediment admixture had no significant effects. In the case of zucchini, our previous study by Wyrwicka *et al*.^79^, demonstrated a strong influence of sewage sludge on the plant metabolic activity. POx activity significantly increased to 149, 265 and 234% of control values in variant amended with 3, 9 and 18 t/ha of sewage sludge. Similarly, GST demonstrated a statistically significant increase being 123, 193 and 199% of the control value for 3, 9 and 18 t/ha of sludge admixture. α-Tocopherol showed increased concentrations in the variants, however significant changes were observed when the highest dose of sewage sludge was applied.

Obtained results demonstrated zucchini to be more sensitive to the applied doses of sewage sludge or sediments than the cucumber proving the validity and necessity for accurate and correct selection of plant species used for phytoremediation purposes.

## Materials and Methods

### Study area

**Figure 8:**
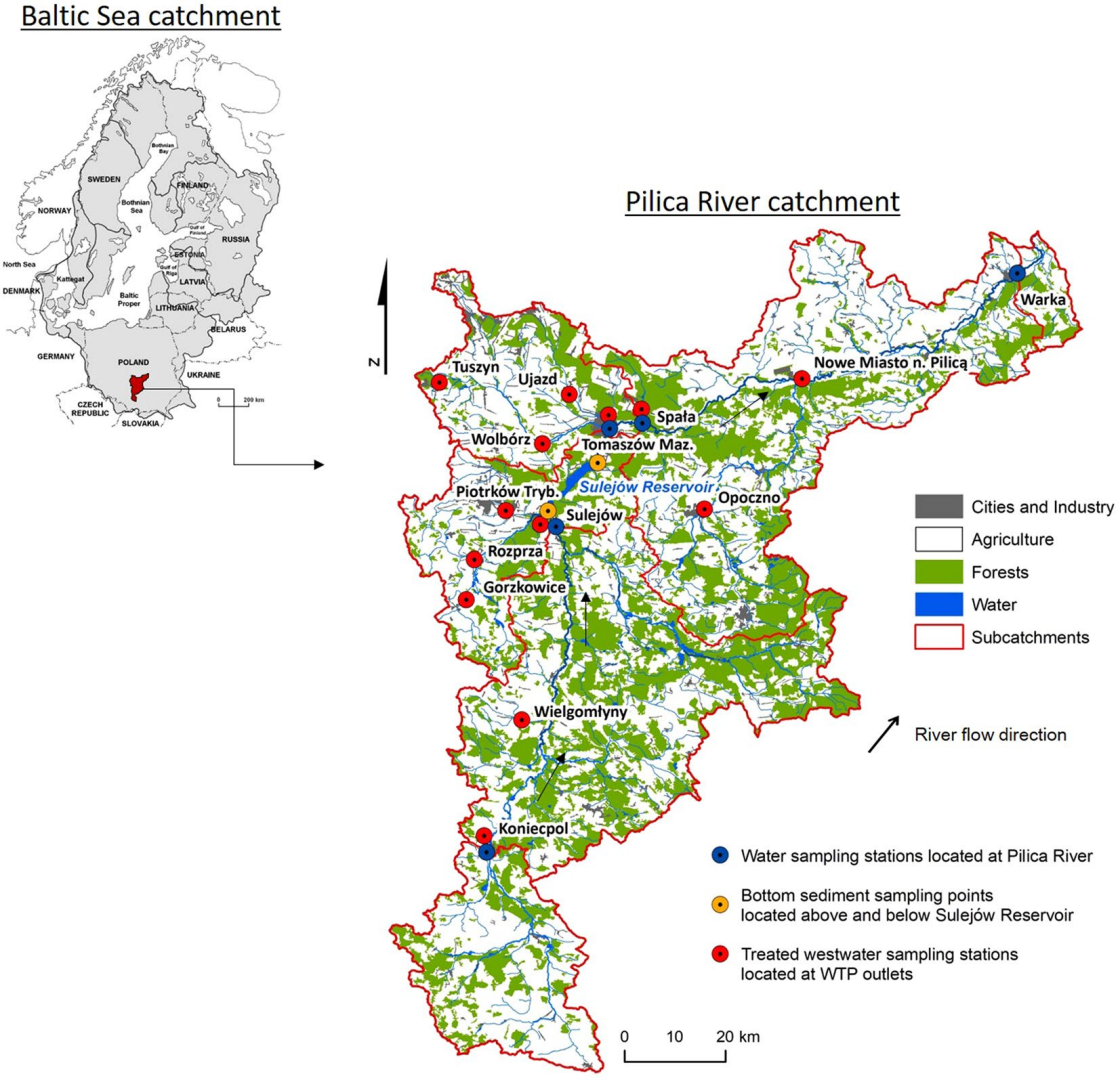
Pilica River catchment (central Poland) with the location of water (blue), sediment (orange) and wastewater (red) sampling stations.

The study was conducted in the Pilica River catchment (Fig. 8). The Pilica River (342 km long) is the longest left-bank tributary of the Vistula River: the largest and one of the most significant rivers in Poland. The middle section of the river includes a lowland dam reservoir – the Sulejów Reservoir^13,20,67^.

The total catchment area of the river is 9,258 km^2^ - the catchment above the reservoir with an area of 4884 km^2^ is mostly agricultural (64.1%), while forests, with a predominance of pine, constitute 26.9%. The catchment below the reservoir is also mainly used for agricultural purposes. Approximately 60% of total catchment area is covered by agriculture, and 31% by forests. Regarding river subcatchments characteristic (Table 1SI, Fig. 8) agriculture is the dominant form of land cover ranging from 51.69% in the case of subcatchment 2 located between Koniecpol and Sulejów sampling points to 71.05% in the case of subcatchment 1 located between source of the river and Koniecpol. Forests cover from 24.2% (subcatchment 4 located between Tomaszów Maz. and Spała sampling points) to 45.4% (subcatchemnt 2 located between Koniecpol and Sulejów). The highest share of industrial areas (0.58%) is noted for subcatchment 3 located between Sulejów and Tomaszów Maz.; while the highest number of urban areas (8.95%) in catchment 4 located between Tomaszów Maz. and Spała (Table 1SI; Fig. 8).

Eleven towns are locat ed along its course, the largest four are Tomaszów Maz. (65,375 inhabitants) with textile, ceramics, machinery, metal and leather industries; Warka (11,035 inhabitants) with strong brewery and fruit processing industries, and Nowe Miasto (3,885 inhabitants)^67,68^.

The catchment encompasses 143 WTPs. These cover 59% of the catchment population, however only 44% are equipped with advanced nutrient removal technology^20^.

The Sulejów Reservoir, constructed in the years 1969–1973, was created by damming the Pilica River in Smardzewice (Central Poland) at km 137. The reservoir served as a water supply until the end of 2012. Currently, the reservoir is used to ensure constant river flow, energy production, reduction of flood waves, recreation and fishing. The length of the reservoir is 25 km, the minimum width in the dam region is 1 km, the maximum width is about 2.2 km, the average depth is 3.3 m. The surface of the reservoir, depending on its filling, is between 19.8 km^2^ and 23.8 km^2^.

The Pilica River and the Sulejów Reservoir have been used for many projects intended to improve water quality by reducing the pollutant loads transported along the river to the reservoir. Together, the river and its catchment comprise the United Nations Educational, Scientific and Cultural Organization (UNESCO) Global Reference Site in Ecohydrology and the Long-Term Socio-Ecological platform of the LTER-Europe Network. Since 2000, the Pilica River has been one of the UNESCO– United Nations Environment Programme (UNEP) demonstration projects developed as part of UNESCO’s International Hydrological Programme (IHP) Ecohydrology Program^82,83^. The Sulejów Reservoir is a part of the Polish National Long-Term Ecosystem Research (LTER) Network and a European LTER site^84^, and serves as a monitoring and research site for the Life + EnvEurope Project LIFE08 ENV/IT/000339 and Life + EKOROB Project LIFE08 ENV/PL/000519^85^. In addition, the Pilica River and Sulejów Reservoir have served as bases for several national projects examining priority hazardous and emerging contaminants^19,62,66–68,86^.

## Samples and Materials

### Field studies - Pilica River catchment

#### Bottom sediments

To determine the role of the Sulejów Reservoir in the transport of the PCDDs/PCDFs and dl-PCBs along the river, the sediment samples were collected in triplicate from the Pilica River at the inflow and ouflow from the Sulejów Reservoir (Fig. 8). Each sample weighed approximately 1 kg (wet weight). The samples were collected using a sediment core sampler. They were then freeze dried, filtered through a 2 mm mesh sieve and mixed to achieve a homogeneous sample; each group of three samples was then mixed in a proportion of 1:1:1 d.w. to obtain a representative sample^67^. Two grams of each representative sample was used for further determination of PCDD/PCDF and dl-PCB content.

#### River water

River water samples were collected during the following conditions:

1. During high water flow (May 2010) in the Pilica River: Under these conditions, water level exceeded the flood alarm level at the water gauge in Sulejów (230 cm, and the corresponding discharge Q = 79.7 m^3^/s) and the discharges at the Sulejów and Spała stations amounted to 216 m^3^/s and 180 m^3^/s (Table 2SI).
2. During serene water flow (September 2010): Under these conditions, the discharges at the Sulejów and Spała stations amounted 38.3 m^3^/s and 54.6 m^3^/s, respectively (Table 2SI).

3) During low water flow (October 2012): The discharges at the Sulejów and Spała stations amounted to 17.6 m^3^/s and 23.9 m^3^/s, respectively (Table 2SI).

Three to five samples were taken using a Teflon sampler from each of five points at bridges along the river continuum, both above and below the reservoir (Fig. 8). The collected water at each point was then transferred into 40.0 L Teflon containers to obtain a single representative sample for each point. Following this, a 5.0 L subsample from each pooled sample was transferred into amber containers and transported to the laboratory in a car refrigerator at a temperature of 4 °C. As the suspended particulate matter constituted less than 1% of the water volume, the unfiltered water was subjected to further analysis of 7 toxic PCDD congeners, 10 PCDF congeners and 12 dl-PCBs. According to United States Environmental Protection Agency guidelines (U.S. EPA)^87^, such samples do not require filtration.

More information regarding the monitoring stations and the physical and chemical parameters during different flow conditions is given in Table 2SI.

#### Treated wastewater

Treated wastewater was collected from the outlets of 14 WTPs discharging into the Pilica River and its tributaries (Fig. 8). The WTPs were divided into three categories according to their population equivalent (p.e.), i.e. the number of adults they can service: small (0–1999 p.e.), medium-sized (2000–9999 p.e), and large (15,000–99,999 p.e.). The WTP characteristics and the physico-chemical parameters of the treated wastewater are depicted in Table 3SI.

Two sets of samples were collected from each WTP in 2010: once during flooding in the spring and again during stable water flow in the summer. The samples were collected directly from the wastewater outflow into the Pilica River or its tributaries as described in *River water*.

### Laboratory experiments – phytoremediation of contaminated sewage sludge and bottom sediments

#### Sewage sludge and bottom sediment

The sewage sludge was obtained from large WTPs located in Lodz, Central Poland, while the bottom sediments were collected from the dam reservoir on the Sokołówka River (Lodz, Central Poland). Both were chosen based on our earlier studies^75,79,81^. All samples were freeze dried for 72 hours and homogenized into small particles with a mortar. They were then used as soil fertilizer for cucumber and zucchini cultivation. The general physico-chemical parameters of the sludge and sediments are depicted in Table 4SI.

#### Experimental setup

All samples were applied to soil at doses of 3, 9 and 18 t/ha. These were chosen to represent the maximum dose of sludge permitted on one occasion per year (3 t/ha) and over three years (9 t/ha), as indicated by Polish Ministry of Environment Regulations (Polish Journal of Laws of 2015, item 257). The highest dose, 18 t/ha, is above the permitted level. These doses constituted, respectively, 1.5%, 4% and 8% of the total dry weight of the sample. Control samples consisted of vegetable potting soil obtained from Hollas Sp. Z o.o. Pasłęk (Poland) with no sludge or sediments. Each treatment variant (Control, 3, 9 and 18 t/ha) was prepared in three replicates in polypropylene pots (capacity ca. 500 cm^3^) and planted with zucchini and cucumber.

Prior to planting, the zucchini (*Cucurbita pepo* L. cv. ‘Atena Polka’) and cucumber (*Cucumis sativus* L. cv. ‘Cezar’) seeds were germinated in Petri dishes for seven days. Seedlings at the same stage of growth and of the same size were then planted into pots containing control soil, soil amended with sewage sludge or soil amended with sediment: nine plants were used per variant - three pots were used for each type of soil, and three seedlings were planted in each pot.

The plants were cultivated for a four-week period in a growth chamber under the following conditions: a temperature of 23+/−0.5 °C, 16-hour light/8-hour dark cycle with 250 µmol m^2^/s photon flux density during the light period and 60% relative humidity.

Subsamples of control soil, soil mixed with sewage sludge or sediment were collected twice during the experiment: at the beginning, to assess the initial concentration of PCDDs, PCDFs and dl-PCBs, and again after four weeks, to assess the removal efficiency of the compounds. Detailed analytical method used for the determination of PCDDs, PCDFs and dl-PCBs is described below.

### Analytical methods

Analysis of 17 toxic congeners of PCDD and PCDF was performed according to U.S. EPA Method 1613^88^. Analysis of 12 dl-PCB was performed according to U.S. EPA Method 1668^80^. Detailed procedure for solid (sediments and soil) and liquid (water and wastewater) samples is described in section “Field studies - Pilica River catchment”.

#### PCDD, PCDF and dl-PCB analysis in sediment and soil samples

The sample of sediment or soil (2 g) was spiked with isotopically-labeled standards (Cambridge Isotopes Laboratories, USA). Extraction was performed with toluene using a Dionex 200 Accelerated Solvent Extractor (150 atm, temperature 175 °C). Potential interference was removed with the use of neutral, acidic and basic silica gels, and elution was performed using hexane (200 mL). The obtained extracts were concentrated to 100 μL, initially by rotary evaporation and then under a gentle stream of nitrogen; the hexane was then replaced with nonane.

PCDD, PCDF and dl-PCB levels were determined using isotope dilution, high-resolution gas chromatography (HRGC) and high-resolution mass spectrometry (HRMS) using an HP 6890 N Agilent Technologies Gas Chromatograph coupled with a high-resolution mass spectrometer (AutoSpec Ultima). As the lock mass (calibration reference), perfluorokerosene was used. The GC equipment was operated in the splitless injection mode. The oven temperature was as follows: 150 °C for 2 min, 20 °C per min to 200 °C (0 min), 1 °C per min to 220 °C (16 min), and 3 °C permin to 320 °C (3 min). The injector temperature was set to 270 °C. The mass spectrometer was operated under positive electron ionization conditions: 34.8 eV electron energy at a resolving power of 10,000 with an ion source temperature of 250 °C. As carrier gas, helium (flow rate 1.60 mL per min) was used.

#### PCDD, PCDF and dl-PCB analysis in water and wastewater samples

Samples consisted of 2.00 L of water or wastewater. These were spiked with isotopically labeled standards (Wellington Laboratories), and extracted with toluene. After evaporation, each sample measured ca. 20.0 mL in volume. The samples were then cleaned overnight in sealed semipermeable polyethylene membrane tubes (wall thickness 80.0 μm) using hexane (100 mL) Obtained dialysate was purified using a silica gel column coated with sulfuric acid and alumina. The final eluate was spiked with precision and recovery solution (20.0 μL) (Cambridge Isotopes Laboratories, USA) and evaporated to 20.0 μL in a gentle stream of nitrogen.

PCDD, PCDF and dl-PCB levels were determined by isotope dilution gas chromatography–mass spectrometry (ID-GC/MS–MS) on a Thermo Scientific GCQ-1100/ Trace2000 system equipped with Xcalibur data acquisition and analysis software. Congeners were separated on a DB5MS J&W capillary column (30.0 m × 0.250 mm i.d.) of 25-μm film and DB17 (30.0 m × 0.250 mm i.d.). A sample of 2.50 μL volume was introduced into a SSL injector at 260 °C. The temperature profile of the GC oven was as follows: 130 °C for 3 minutes, 50 °C per min to 180 °C 2 °C per min to 270 °C, and 20 °C per min to 300 °C (5 min). The result uncertainty was expressed as extended measurement uncertainty for k = 2 at a confidence level of 95%.

### QA/QC

The glassware used in the field and laboratory were cleaned with detergent, rinsed with ultrapure water, and heated overnight at 450 °C. The glassware was additionally cleaned with acetone and hexane before use. The Teflon containers used in the field were also cleaned with detergent, rinsed with ultrapure water, and then with acetone and hexane.

QA/QC was ensured with the use of certified calibration standards. Each analytical batch included a method blank, a matrix spike and replicate samples. Artifacts were assessed using a reagent blank while precision was confirmed by duplicate analyses. Samples spiked with PCDDs, PCDFs or dl-PCBs were used to determine recoveries and to verify accuracy. Additionally, BCR-615 fly ash Low level, CRM-490 fly ash Municipal waste and 1939a Polychlorinated Biphenyls in River Sediment A were used as standard reference materials.

Detection levels for sediment and soil samples ranged from 0.11 to 0.84 pg/kg for PCDDs, PCDFs, and from 0.026 to 0.111 pg/kg for dl-PCBs. Detection levels for water and wastewater samples ranged from 0.44 to 1.41 pg/L for PCDDs/PCDFs, and 0.40 to 1.90 pg/L for dl-PCBs.

## Conclusions

Our findings, derived from field studies, outline the problems associated with PCDD, PCDF and dl-PCB pollution of both the Pilica River and Sulejów Reservoir, with the determining role of hydrological conditions prevailing in the river itself and its catchment on the quoted concentrations. The values recorded during the flood season were around 46% higher than observed during the period of stable flow. Also, the loads of the studied compounds transported during the flood increased from 5- to 12-fold in the case of the total concentration and from 23- to 60-fold in the case of Toxic Equivalency (TEQ). The primary cause of such significant differences is the form of the hydrological conditions prevailing in the river itself and its catchment. The obtained results highlighted also the positive role of Sulejów Reservoir in reduction of the transported PCDD, PCDF and dl-PCB during the study time. In this case the obtained results demonstrated decreases in the studied compounds concentrations ranging from 17% to 83% for total values, and from 33% to 79% for TEQ values. Nevertheless, this positive role may change over time e.g. and under reduced pollutant load or turbulent flow the sediments accumulated at the bottom of reservoir may act as a source of the studied compounds.

The analysis demonstrated also that all 14 studied WTPs do not purify wastewater in a sufficient extent. The highest PCDD, PCDF and dl-PCB concentrations were observed in the small WTPs, while large ones showed the lowest values. However, an opposite situation was observed when the obtained values were calculated into loads discharged to the Pilica River and its tributaries. In this case, the highest loads were generated by large WTPs, while small WTPs, despite high concentration, discharged the smallest loads of studied compounds into the recipients.

Regarding WTPs, in addition to discharging PCDD, PCDF and dl-PCB into the aquatic environment, they also produce sewage sludge as the end product of the treatment process. There is also a need to utilize river and reservoir sediments contaminated with PCDD, PCDF and dl-PCB as an effect of their discharge via i.e. WTP outlets.

Both, the contaminated sewage sludge and sediments served as the basis for the laboratory experiments aimed at assessing the effectiveness of cucurbits in removing PCDD, PCDF and dl-PCB. The obtained results revealed that these pollutants may be removed by using the contaminated sewage sludge and sediments as plants fertilizer. Application of cucumber (*Cucumis sativus* L.) and zucchini (*Cucurbita pepo* L.) resulted in a decrease in PCDD, PCDF and dl-PCB TEQ concentrations by an average of 64% (for cucumber) and 69% (for zucchini) in soil fertilized with sludge and 52 and 51% (for cucumber and zucchini, respectively) in soil fertilized with sediments, demonstrating a positive influence of the applied plants on the soil quality.

The use of both field and laboratory studies, presented in the article, constitutes an example of a holistic, ecohydrological approach to addressing the problem of PCDD, PCDF and dl-PCB pollution within the river catchment (Fig. 1SI). Insight into the role of complex interactions occurring in the catchment, allows enhancement in our understanding of hydrological and ecological processes to protect water resources. Such approach is intended to secure the ecosystem well-being for future generations and in this way brings benefits for society.

## Supplementary information

Supporting Information
