# Supplementary material for: An ecohydrological approach to the river contamination by PCDDs, PCDFs and dl-PCBs – concentrations, distribution and removal using phytoremediation techniques
Source: Sci Rep. 2019 Dec 17;9:19310. doi: 10.1038/s41598-019-55973-3 (PMC6917701; doi:10.1038/s41598-019-55973-3)
Supplement: Supplementary file 1 — Supporting Information [file 41598_2019_55973_MOESM1_ESM.docx]

**Supplementary Information**

**An ecohydrological approach to the river contamination by PCDDs, PCDFs and dl-PCBs – concentrations, distribution and removal using phytoremediation techniques**

Urbaniak M.^1,2,3^, Kiedrzyńska E.^1,2^, Wyrwicka A.^4^, Zieliński M.^5^, Mierzejewska E.^2^, Kiedrzyński M.^6^, Kannan K.^3^, Zalewski M.^1,2^

^1^ European Regional Centre for Ecohydrology of the Polish Academy of Sciences, Tylna 3, 90-364 Lodz, Poland

^2^ Department of Applied Ecology, Faculty of Biology and Environmental Protection, University of Lodz, Banacha 12/16, 90-237 Lodz, Poland

^3^ Wadsworth Center, New York State Department of Health, Empire State Plaza, Albany, NY 12201-0509, USA

^4^ Department of Plant Physiology and Biochemistry, Faculty of Biology and Environmental

Protection, University of Lodz, Banacha 12/16, 90-237 Lodz, Poland

^5^ Nofer Institute of Occupational Medicine, Teresy 8, 91-348 Lodz, Poland

^6^ Department of Geobotany and Plant Ecology, Faculty of Biology and Environmental Protection, University of Lodz, Banacha 12/16, 90-237, Lodz, Poland.

Corresponding author: Magdalena Urbaniak, email: m.urbaniak@unesco.lodz.pl

**Table 1. SI.** Characteristic of the Pilica River subcatchments land cover between individual monitoring profiles (calculated from CORINE-2006)

| **Type of land cover** | **Subcatchment** | | | | |
| --- | --- | --- | --- | --- | --- |
|  | **Subcatchment 1** | **Subcatchment 2** | **Subcatchment 3** | **Subcatchment 4** | **Subcatchment 5** |
|  | (between river source and Koniecpol) | (between Koniecpol and Sulejów) | (between Sulejów and Tomaszów Maz.) | (between Tomaszów Maz. and Spała) | (between Spała and Warka) |
| Urban [%] | 1.51 | 1.99 | 5.97 | 8.95 | 3.38 |
| Industrial [%] | 0.03 | 0.12 | 0.58 | 0.32 | 0.16 |
| Agriculture [%] | 71.05 | 51.69 | 60.38 | 66.15 | 62.36 |
| Forests [%] | 27.02 | 45.43 | 30.22 | 24.24 | 33.34 |
| Water and wetlands [%] | 0.39 | 0.78 | 2.85 | 0.35 | 0.76 |
| **Total sub-catchment area [km^2^]** | 100 | 100 | 100 | 100 | 100 |

**Table 2. SI.** Characteristics of river monitoring stations and physical and chemical parameters of the Pilica River (central Poland) water during high (2010 yr), stable (2010 yr), and low (2012 yr) water flow.

| River and catchment characteristic in monitoring stations | **Station name** | **Koniecpol** | | | **Sulejów** | | | **Tomaszów Mazowiecki** | | | **Spała** | | | **Warka** | | |
| --- | --- | --- | --- | --- | --- | --- | --- | --- | --- | --- | --- | --- | --- | --- | --- | --- |
|  | River km from the estuary | 261.7 | | | 161.3 | | | 128.5 | | | 119.4 | | | 15 | | |
|  | Drainage area [km^2^] | 1,088.7 | | | 3,935.7 | | | 4,991.5 | | | 5,967.3 | | | 9,076.6 | | |
|  | Hydrological situation (water flow) | High | Stable | Low | High | Stable | Low | High | Stable | Low | High | Stable | Low | High | Stable | Low |
|  | Discharge [m^3^ /s] | - | - | - | 216 | 38.3 | 17.6 | 176.4* | 41.6* | 24.0* | 180 | 54.6 | 23.9 | - | - | - |
|  | Outflow [mln m^3^ /day] | - | - | - | 18.7 | 3.3 | 1.52 | 15.2* | 3.59* | 2.07* | 15.6 | 4.11 | 2.06 | - | - | - |
| Physical parameters | Temperature [°C] | 11.8 | 12.7 | 17.2 | 11.4 | 14.3 | 17.6 | 13.5 | 14.5 | 19.4 | 14.1 | 14.1 | 19.3 | 17.3 | 14.6 | 22.8 |
|  | Disolvent oxigen [mg /L] | 7.6 | 7.5 | 8.1 | 9.1 | 8.1 | 7.9 | 7.1 | 7.6 | 6.8 | 7.5 | 7.4 | 7.2 | 7.5 | 6.0 | 8.9 |
|  | pH | 7.7 | 7.9 | 8.0 | 7.4 | 8.0 | 8.0 | 7.8 | 7.9 | 7.5 | 7.7 | 5.1 | 7.1 | 7.9 | 5.3 | 6.7 |
|  | Conductivity | 308 | 468 | 406 | 171 | 384 | 303 | 339 | 177 | 292 | 368 | 371 | 341 | 337 | 790 | 331 |
|  | Mineral matter [mg /L] | 19 | 12 | 2 | 2 | 1 | 6 | 3 | 1 | 1 | 3 | 1 | 1 | 1 | 1 | 4 |
|  | Organic matter [mg /L] | 6 | 2 | 3 | 3 | 1 | 6 | 3 | 2 | 5 | 3 | 2 | 4 | 1 | 1 | 7 |
|  | Total matter [mg dm^-3^] | 24 | 14 | 5 | 5 | 2 | 12 | 6 | 3 | 7 | 6 | 3 | 6 | 2 | 2 | 11 |
|  | % content of mineral matter | 76 | 85 | 36 | 39 | 63 | 50 | 54 | 43 | 21 | 45 | 28 | 21 | 33 | 46 | 39 |
|  | % content of organic matter | 24 | 15 | 64 | 61 | 37 | 50 | 46 | 57 | 79 | 55 | 72 | 79 | 67 | 54 | 61 |
| Chemical parameters | Total phosphorus (TP) [mg /L] | 1.74 | 1.13 | 0.24 | 0.27 | 1.21 | 0.16 | 0.06 | 0.65 | 0.32 | 0.18 | 0.55 | 0.18 | 0.60 | 1.15 | 0.17 |
|  | Soluble Reactive Phosphorus (SRP) [mg /L] | 1.69 | 1.10 | 0.14 | 0.23 | 0.14 | 0.09 | 0.02 | 0.12 | 0.13 | 0.10 | 0.16 | 0.17 | 0.10 | 0.19 | 0.02 |
|  | Total Nitrogen (TN) [mg /L] | 11.09 | 12.12 | 2.20 | 3.47 | 5.16 | 1.04 | 3.37 | 3.11 | 0.21 | 5.21 | 4.03 | 1.12 | 5.54 | 3.92 | 0.38 |
|  | Nitrite [mg /L] | 0.47 | 0.05 | 0.00 | 0.03 | 0.02 | 0.00 | 0.04 | 0.04 | 0.00 | 0.03 | 0.03 | 0.00 | 0.04 | 0.00 | 0.00 |
|  | Nitrates [mg /L] | 10.57 | 11.99 | 1.85 | 3.42 | 4.73 | 0.92 | 3.20 | 2.93 | 0.13 | 5.03 | 3.89 | 0.37 | 5.49 | 3.88 | 0.37 |
|  | Ammonium [mg /L] | 0.05 | 0.07 | 0.35 | 0.02 | 0.41 | 0.11 | 0.13 | 0.14 | 0.09 | 0.14 | 0.11 | 0.74 | 0.01 | 0.03 | 0.01 |
|  | Fluoride [mg /L] | 0.08 | 0.17 | 15.59 | 0.18 | 0.18 | 12.99 | 0.15 | 0.22 | 14.08 | 0.18 | 0.22 | 34.46 | 0.17 | 0.22 | 19.08 |
|  | Chloride [mg /L] | 15.05 | 13.18 | 0.00 | 5.87 | 11.34 | 0.02 | 11.73 | 10.41 | 0.00 | 23.32 | 12.35 | 0.00 | 12.69 | 13.47 | 0.00 |
|  | Bromides [mg /L] | 0.01 | 0.01 | 20.02 | 0.01 | 0.01 | 25.01 | 0.01 | 0.03 | 30.59 | 0.02 | 0.01 | 35.73 | 0.01 | 0.01 | 35.89 |
|  | Sulfur [mg /L] | 21.83 | 28.33 | 0.01 | 21.85 | 23.67 | 0.00 | 31.09 | 23.94 | 0.00 | 49.65 | 27.71 | 0.00 | 33.98 | 29.62 | 0.00 |
|  | Lithium [mg /L] | 0.00 | 0.00 | 9.09 | 0.00 | 0.00 | 6.23 | 0.00 | 0.00 | 6.75 | 0.00 | 0.00 | 17.12 | 0.00 | 0.00 | 12.77 |
|  | Sodium [mg /L] | 2.97 | 4.65 | 10.29 | 2.43 | 5.21 | 10.32 | 5.18 | 5.06 | 12.69 | 5.76 | 6.94 | 14.35 | 6.14 | 8.72 | 14.30 |
|  | Potassium [mg /L] | 5.60 | 7.50 | 0.93 | 4.45 | 6.77 | 0.86 | 7.50 | 6.76 | 1.30 | 8.20 | 7.62 | 1.21 | 9.24 | 9.45 | 1.11 |
|  | Magnesium [mg /L] | 1.00 | 1.29 | 65.88 | 0.84 | 0.73 | 61.97 | 1.00 | 0.82 | 64.36 | 2.56 | 0.92 | 66.65 | 1.25 | 1.03 | 51.02 |
|  | Calcium [mg /L] | 55.71 | 97.53 | 0.02 | 29.72 | 74.33 | 0.13 | 56.16 | 65.48 | 1.69 | 56.24 | 67.06 | 0.02 | 48.82 | 67.54 | 1.88 |

d.n.a – data not available, *- discharge and outflow taken from nearest located gauge station in Smardzewice

**Table 3. SI**. Characteristics of wastewater treatment plants located in the Pilica River catchment, central Poland, and the physico-chemical parameters of the treated wastewater.

| **WTPs size** | **WTP location** | **WTP size class** | **WTP population equivalent** | **Daily average outflow**  **[m^3^/day]** | **Treatment steps** | **Nutrients concertations** | | **Recipient river** |
| --- | --- | --- | --- | --- | --- | --- | --- | --- |
|  |  |  |  |  |  | **Average [mg/L]** | **Max [mg/L]** |  |
| Small | Koniecpol | I | 600 | 100 | secondary | TP  9.24  TN 62.06 | TP 68.31  TN 295.42 | **Pilica** |
|  | Wielgomłyny | I | 1000 | 200 | secondary |  |  | Biestrzykowka |
|  | Gorzkowice | I | 700 | 224 | secondary |  |  | Prudka |
|  | Rozprza | I | 500 | 107 | secondary |  |  | Dabrowka |
|  | Ujazd | I | 1500 | 300 | secondary |  |  | Piasecznica |
|  | Wolbórz | I | 800 | 241 | secondary |  |  | Moszczanka |
|  | Spała | I | 350 | 130 | secondary |  |  | Gac |
| Medium-sized | Sulejów | II | 7500 | 870 | secondary | TP  4.08  TN 33.67 | TP 13.63  TN 65.14 | **Pilica** |
|  | Nowe Miasto | II | 2583 | 1000 | secondary |  |  | **Pilica** |
|  | Tuszyn | II | 4000 | 640 | secondary* |  |  | Wolborka |
| Large | Tomaszów Maz. | IV | 80000 | 10050 | secondary | TP  1.97  TN 45.97 | TP 6.97  TN 139.41 | **Pilica** |
|  | Warka | IV | 99000 | 9900 | secondary* |  |  | **Pilica** |
|  | Piotrków Tryb. | IV | 80000 | 14541 | secondary |  |  | Moszczanka |
|  | Opoczno | IV | 75000 | 5127 | secondary |  |  | Drzewiczka |

* with advanced nutrient removal; TP- Total Phosphorus; TN- Total Nitrogen

**Table 4. SI**. Characteristics of physical and chemical parameters of sewage sludge and sediments used in laboratory phytoremediation experiment.

| **Contaminant** | **Mean content** | |
| --- | --- | --- |
|  | **Sewage sludge** | **Sediments** |
| pH | 7.91 | 7.38 |
| Organic matter % | 64.4 | 32.9 |
| Nitrogen (N) % | 5.04 | 9.69 |
| Phosphorus (P) [mg/kg] | 2670 | 6070 |
| Magnesium (Mg) [mg/kg] | 5100 | 7040 |
| Sodium (Na) [mg/kg] | n.a | 1520 |
| Potassium (K) [mg/kg] | 2400 | 1550 |
| Calcium (Ca) [mg/kg] | 28600 | 9700 |
| Iron (Fe) [mg/kg] | n.a. | 4150 |
| Cadmium (Cd) [mg/kg] | 4.45 | 1.2 |
| Chromium (Cr) [mg/kg] | 188 | 10.58 |
| Copper (Cu) [mg/kg] | 452 | 32.0 |
| Lead (Pb) [mg/kg] | 82.3 | 12.6 |
| Nickel (Ni) [mg/kg] | 92.1 | 8.65 |
| Zinc (Zn) [mg/kg] | 1813 | 136 |
| Lead (Hg) [mg/kg] | 3.75 | 0.14 |

n.a.- not analyzed

**
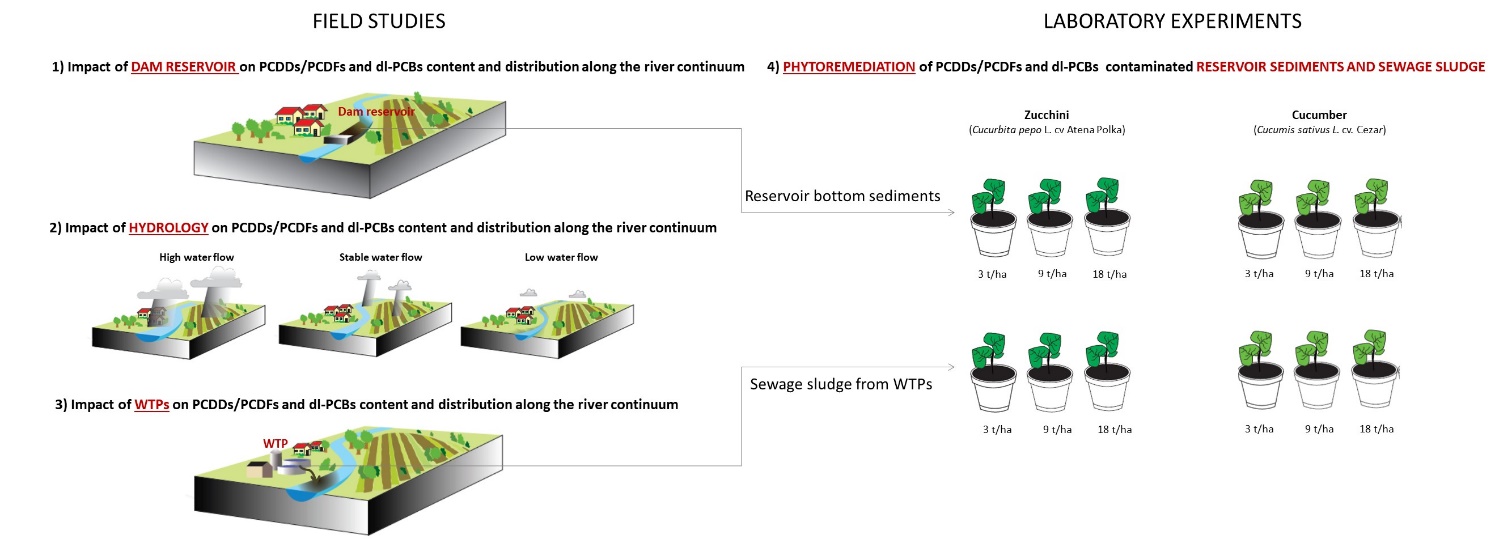
**

**Fig. 1SI.** Integration of field and laboratory studies as an ecohydrological approach to addressing the PCDDs, PCDFs and dl-PCBs pollution within the catchment scale


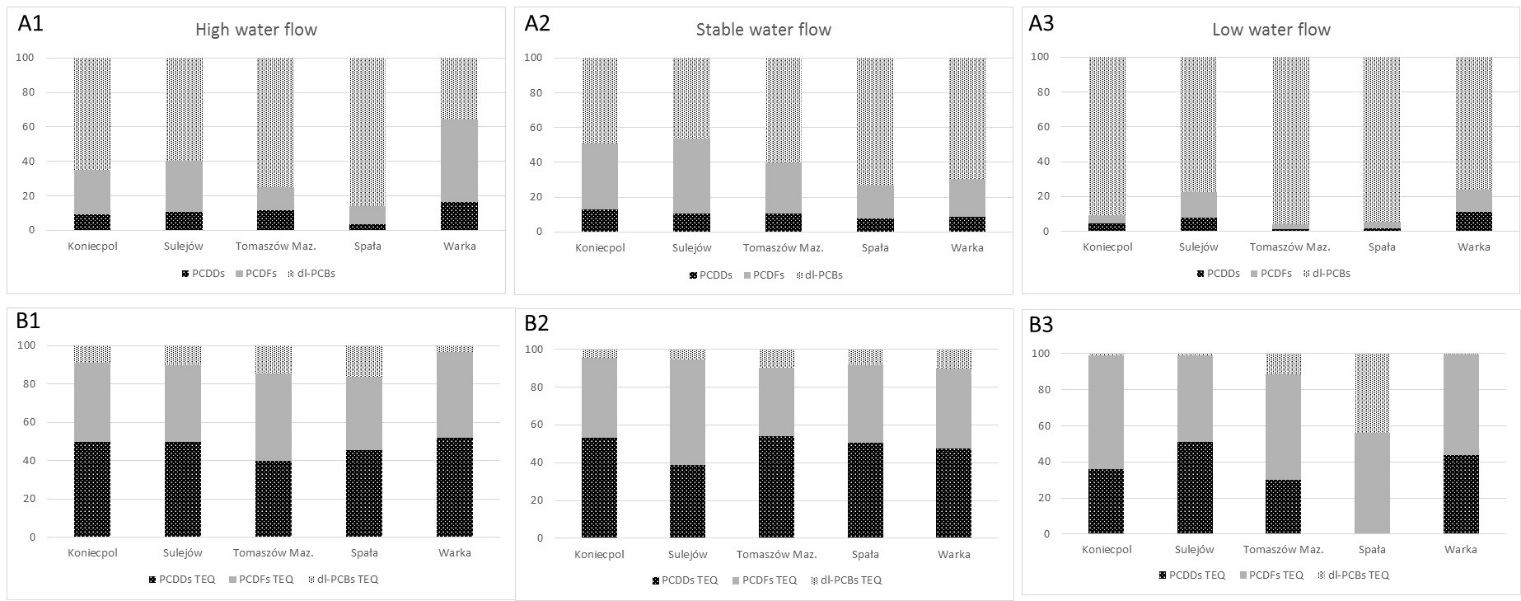


**Fig. 2. SI**. PCDDs, PCDFs and dl-PCBs patterns in the Pilica River (Poland) water samples collected at high, stable and low river flow conditions along the river continuum: A1-A3 contribution of PCDDs, PCDFs and dl-PCBs for the total concentration; B1-B3 – contribution of PCDD TEQ, PCDF TEQ and dl-PCB TEQ in the total TEQ concentration.
